# The sentry and the architect: functional specialization of two jasmonate-synthesizing enzymes in rice

**DOI:** 10.1093/plcell/koag238

**Published:** 2026-08-06

**Authors:** Ved Prakash

**Affiliations:** Assistant Features Editor, The Plant Cell, American Society of Plant Biologists; Department of Plant Pathology, College of Food, Agriculture, and Environmental Sciences, The Ohio State University, 1680 Madison Ave., Wooster, OH 44691, United States

The phytohormone jasmonic acid (JA) participates in a variety of roles in plants, from growth and development to defense against attacking insects/necrotrophic pathogens (Cai et al. 2014; Wasternack and Song 2017; Howe et al. 2018). JA is not the bioactive signal itself. Instead, it is conjugated with amino acids (AA), such as isoleucine (Ile), to result in JA-Ile as bioactive form (Howe et al. 2018). This conversion from JA to JA-AA is catalyzed by a family of enzymes called JASMONATE RESISTANT (JAR) amido synthetases (Howe et al. 2018). Researchers have long wondered how a single hormone coordinates different biological outputs, such as seedling growth and flowering, and acute herbivore attack.

In this issue, **Lei-Lei Li and colleagues** (Li et al. 2026) show that rice (*Oryza sativa*) uses distinct JAR enzymes to channel jasmonate signaling toward herbivore defense and spikelet development (see Fig. 1). They used CRISPR-Cas9 to edit *JAR* genes and generated a set of loss-of-function mutant plants to show how these enzymes separately function in growth and defense.

**Figure 1:**
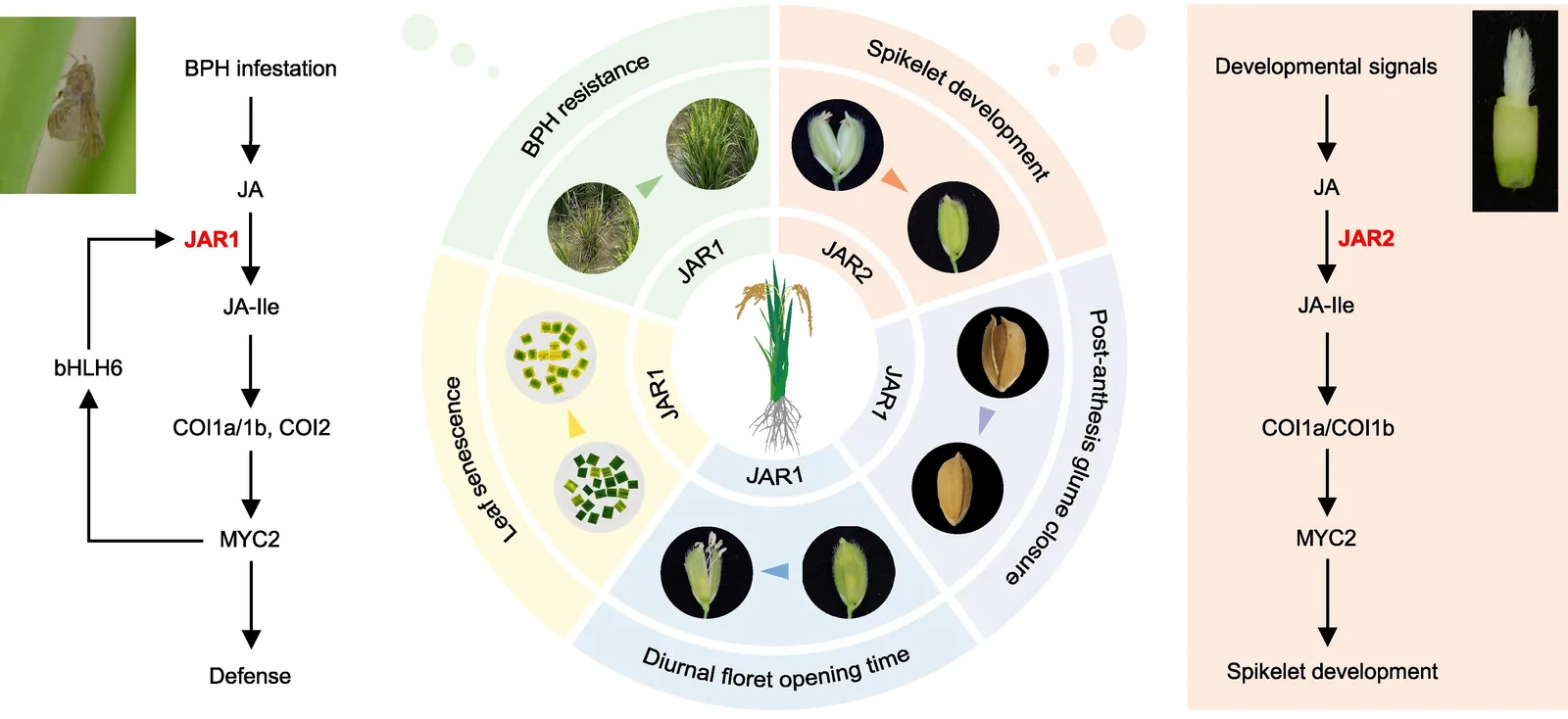
Schematic representation of the role of *JAR1* and *JAR2* in rice growth and defense. *JAR1* is involved in defense against BPH resistance, leaf senescence, post anthesis glume closure, and diurnal floret opening time, while *JAR2* controls spikelet development. Adapted from Li et al. (2026), Fig. 8.

The rice genome contains 3 *JAR* genes: *OsJAR1*, *OsJAR2*, and *OsJAR3*. The authors found that *OsJAR3* is root-enriched and biochemically attenuated, and mutant analysis suggests that it contributes minimally to rice growth and defense. They found that OsJAR3 has alanine at position 335 instead of the conserved glycine in motif 2, which severely weakens its enzymatic efficiency. In comparison, OsJAR1 and OsJAR2 are the main enzymes involved in growth and defense. Together, OsJAR1 and OsJAR2 function redundantly to synthesize active JA-Ile for normal seedling growth. *jar1 jar2* double mutants lacked detectable basal JA-Ile and ultimately died at the tillering stage, indicating that OsJAR1 and OsJAR2 redundantly sustain vegetative growth.

However, as the plant matures, *OsJAR1* and *OsJAR2* diverge in their function and take on separate and specialized roles to help separate developmental and defense outputs. When piercing-sucking brown planthoppers (BPH) attack the rice plant, *OsJAR1* is induced. The insect attack triggers JA-Ile synthesis, which activates the MYC2-bHLH6 transcriptional cascade. The bHLH6 binds to the *OsJAR1* promoter to induce *OsJAR1*'s transcription. The OsJAR1 protein, in turn, synthesizes active JA-Ile, creating a positive feedback loop. The increased level of JA-Ile triggers the plant to produce anti-BPH chemical compounds called phenolamides to promote defense against BPH by altering egg hatching duration and reducing rice susceptibility under natural field conditions. At later stages of the plant development, OsJAR1 also functions in leaf senescence, diurnal floret opening time, and post-anthesis glume closure (see Fig. 1).

In contrast, *OsJAR2* is not induced during insect attack and is not required for defense. Instead, it is synthesized spatiotemporally during the very early stages of inflorescence differentiation. Interestingly, OsJAR2 produces JA-Ile without involving the MYC2-bHLH6 feedback amplification loop and regulates development and reproductive organ-related genes via the canonical OsCOI1-MYC2 signaling pathway to ensure that floral structures form with the correct shape, numbers, and layout (see Fig. 1).

By using 2 different genes from the *JAR* gene family, rice plant creates a division of labor for fighting herbivores and a controlled spatiotemporal signaling pathway for floral development. The findings from this study illustrate functional specialization of closely related enzymes, demonstrating how rice plants use a clear division of labor to independently coordinate defense responses and development.

## Recent related articles in *The Plant Cell*

- Luo et al., (2026) identified the *PMR4* gene as a host susceptibility factor and a potential target for powdery mildew and clubroot resistance in *Brassica napus*.
- Song et al., (2026) found that JA induces death of spongy mesophyll cells in the initial stage of leaf senescence and is mediated by the *CmNAC055* and *CmNAC087*.
- Li et al. (2024) showed how the nucleotide pathway and JA metabolism are connected. They found that *par1* mutants accumulate high JA level which might affect maize growth.

## Data Availability

No new data were generated or analysed in support of this research.
